# Hospital and Institutional News

**Published:** 1921-03-12

**Authors:** 


### HOSPITAL AND INSTITUTIONAL NEWS.

### DANGEROUS DRUGS DAY BY DAY.

The Home Secretary stated last week in the House of Commons that he had agreed to receive a deputation from the pharmaceutical societies regarding the Dangerous Drugs Act Draft Regulations. He also said that he appreciated the importance, in relation to the proper care of stock, that farmers should have in their possession mixtures and other drugs for immediate use in the case of sudden illness of their stock; he had given the fullest consideration to the views of Scottish agriculturists on this point. Mr. F. A. Hocking tells us that the scheme which he referred to the *Pharmaceutical Journal* for the regulation of dangerous drugs in hospitals has no connection with that put forward by Mr. Billington. We look forward with interest to particulars of the scheme with which Mr. Hocking's name is associated. A meeting of various medical and trade organisations was held last week, under the presidency of Mr. E. B. Turner, F.R.C.S., and as a result a request has been made to Mr. Shortt to appoint an expert committee to advise on the regulations. In the letter to the Home Secretary the belief is expressed that he has "no conception of the strong resentment which has been caused by the publication of the draft Regulations without previous effective consultation with bodies that are deeply and legitimately interested in dealings with dangerous drugs."

### AN APPEAL TO THE HOME SECRETARY.

From time to time Lambeth Board of Guardians has tried to alter the present police arrangements, by which people meeting with accidents in the street are taken to the nearest hospital, although a Poor-law infirmary may be much nearer. Much valuable time is wasted by this method, but, unfortunately, the Lambeth Guardians have been unable to persuade the Chief Commissioner of Police to alter his instructions. Some time ago a woman was knocked down in Brooke Street, Lambeth, close to the infirmary, but, instead of allowing her to be taken into the infirmary, the police, acting under their instructions, sent for the London County Council ambulance, and took the patient to St. Thomas's Hospital. Now a more serious case has occurred. A woman, sixty-six years old, was knocked down by a motor-car at nine o'clock at night, sustaining severe injuries. She was taken to King's College Hospital, but was afterwards sent to Lambeth Infirmary, where she arrived a few minutes after midnight. The Guardians have asked for the observations of the staff at King's College Hospital on this case, and have now decided to do what they should have done long ago, and that is to appeal to the Home Secretary to instruct the police to take patients injured in street accidents to the nearest institution, be it either a Poor-law infirmary or a public hospital. What has Mr. Edward Shortt to say to this?

### MUNICIPAL HELP FOR ST. THOMAS'S HOSPITAL.

The Mayor of Lambeth has responded to the appeal of the governors of St. Thomas's Hospital for help to clear off the debt of £100,000 and to place the finances on a sound footing. In this effort he has been promised the support of a large number of the leaders of social and political life in the borough, also that of large employers of labour. The proposal is to bring the financial needs of the hospital to the notice of the huge working-class population in Lambeth with a view to obtaining their monetary help. The hospital has a great call not only upon Lambeth, but also upon the neighbouring boroughs of Southwark and Bermondsey, for it was on the borders of the two boroughs that it was founded over seven centuries ago, and to-day many patients are admitted from Southwark and Bermondsey. Southwark has no large hospital within its boundaries, but, wedged between Bermondsey and Lambeth, it calls upon either St. Thomas's or Guy's to attend to its sick. Why not extend the appeal to Southwark, full, as it is, of warehouses and manufacturing firms, all of whom are only too ready to call upon the resources of the hospital in time of need? An appeal to them should meet with a hearty response. Perhaps a request for help to the Mayor of Southwark, an energetic man with wide sympathies, would meet with success either on behalf of St. Thomas's or Guy's.

### KNOWLEDGE OF GREEK OR GERMAN BARRED.

Litigation in the Probate Court has delayed the granting of probate of the will of the late Dr. Charles Arthur Mercier, the well-known alienist, who died on September 1, 1919. Under this will Dr. Mercier directed that the residue of his estate, after payment of bequests to relatives and friends, should remain and accumulate until it amounted to £20,000, when the executor is to offer it to the London or other University for the endowment of a professorship of "Rational Logic and Scientific Method," for teaching students not what Aristotle or anyone else thought about reasoning, but to think and reason for themselves. A condition of the will was that in selecting the professor preference should be given to the candidate "who knows neither Greek nor German." The estate is valued at £4,899, and unless the development of the Wolfryn process for the electrification of seeds for wheat-growing, in which the estate has an interest, proceeds rapidly, it is probable that the appointment of the professor who is neither on the modern nor the classical side will be delayed for a few years.

### A FRIENDLY LAWSUIT.

Mr. Justice Lawrence had before him recently a summons to determine the allocation of the surplus assets of the Welsh Hospital, Netley, Fund. When the hospital was wound-up in 1919 there was in hand about £10,000 in War Loan Stock and cash, and the trustees put forward a scheme to give these assets to the University of Wales to encourage the study of medicine and surgery by persons of Welsh nationality. To put the matter in order, a defendant, in the person of a subscriber to the fund, obligingly came forward. The judge deferred his decision pending further inquiry. He liked the educational proposal, but said that he would prefer also to consider a scheme more immediately applied for the benefit of those still sick and wounded. The Attorney-General and the trustees are to confer further and to report to him in chambers.

### UNEMPLOYMENT AND SEAMEN'S HOSPITALS.

The slump in freight rates has made shipping so unprofitable that hundreds of vessels are laid up in the ports of the United Kingdom and their crews paid off. This is having its effect upon some hospitals, especially those for seamen. This state of affairs was commented on by Mr. L. R. Turnbull, when presiding at the sixteenth annual meeting of the Royal Hamadryad Seamen's Hospital, Cardiff. He pointed out that the coming year would, therefore, be very trying for the institution, and made a strong appeal for increased contributions. The Hamadryad's income showed a slight increase last year, but this was more than swallowed up by the enhanced cost of materials, and the total expenditure showed an advance of £1,142 over the preceding twelve months. The hospital, however, is in the fortunate position of having an excess of income over expenditure, due, doubtless, to the economical manner in which it is worked, the cost per occupied bed per day being apparently only 7s. 10½d. An interesting feature of the annual meeting was the tribute paid to Miss M. W. Corbett, Miss Gladys Corbett, and Miss Mabel Stewart for their continued and valuable services as honorary nurses. The whole question of unemployment and hospitals needs to be considered, but it is not easy to collate the facts concerning each type of institution, and generalisations are not too helpful at such a time.

### HOSPITAL PROGRESS IN ICELAND.

Just as some of the best books fail to get written because their authors lack tenacity for their task, so many excellent periodicals continue almost unknown through the fault of—is it their editors or the public? There is a good deal of unexpectedly interesting reading, for example, in the February number of *Toilers of the Deep*, the monthly magazine of the Royal National Mission to Deep Sea Fishermen. He who wants to know what a *coper* was and how it was driven from the North Sea will find the information in the opening article. But it is not this, nor the generously illustrated article on Icelandic life, to which we wish now to draw special attention. We are immediately indebted to the paper for the account by Dr. J. L. Nisbet, the Society's medical missionary in Iceland, of the steps being taken in Iceland to establish a National General Hospital. The women of Iceland seem to have supported the building fund energetically, and the appearance of these workers can be studied in some attractive photographs. Hitherto, Mr. Nisbet writes, small hospitals belonging to the towns and villages were to be found around the coast. But the present plan is for a modern general hospital to be associated with the medical school at Reykjavik, near which "a first-rate sanatorium for consumptives was founded in 1911. We also learn, if too briefly, of a large isolation hospital for lepers, and a modern hospital for mental disease. We hope that Mr. Nisbet will return to this part of his subject in greater detail, for is there anywhere which seems so remote as Iceland—the land of all others, he affirms, where there are more poets than anywhere else? Was that why William Morris used to go there?

### DR. W. T. GRENFELL ON LABRADOR.

The Home for Little Children at St. Anthony, Labrador, is the subject of an interesting article by Dr. Wilfred T. Grenfell in last month's issue of *Toilers of the Deep*. We learn, for instance, that "the Northern Eskimo, unable to make provision for their enfeebled old folk, just leave the helpless aged to perish of exposure." Whatever the young eugenist might think of this plan, the elderly eugenist fallen into distress would probably dissent from it. Consequently Dr. Grenfell, whose autobiography entitled "A Labrador Doctor" lately appeared, has been busy trying to raise an endowment fund for the institution in which he is interested, both in this country and in Canada. Dr. Harry Paddon, of Oxford, and Dr. Charles Curtis, of Harvard, have assisted him. His article is interesting, not only for its references to Labrador and the hospital problems there, but because of the conclusions which he has formed after a long experience in collecting funds for voluntary bodies. He has learnt, he says, that "problems of every kind are the dignity of life," and certainly the reward of such work is no less in the struggle than in the victory for which the struggle is made. If we compare the mentality of an official of the Treasury with that of a financial secretary to a voluntary hospital, who shall say that the latter's outlook is not the wider and more generous? Voluntary work, because it makes the most demands upon a man, brings out the best that is in him. He learns, too, as public officials often fail to learn, that, in Dr. Grenfell's words, "the Christian has no right to anxiety," a lesson that makes alike for judgment and cheerfulness.

### THE NATIONAL RELIEF FUND AND WALSALL HOSPITAL.

Mr. F. J. Cotterell, the Chairman of the Walsall General Hospital, said at the annual meeting that it was a matter for satisfaction that the ordinary income last year showed an increase of 20 per cent. There was also an increase in workpeople's subscriptions of over £1,600 compared with 1919. This, he thought, was a striking proof that their supporters were in favour of the continuance of the voluntary system. The hospital has not been so fortunate as to receive any grant from the National Relief Fund, although an application had been made. Much disappointment was felt by the Committee, especially as the year 1920 closed with a deficit of £7,200. Apparently the view taken was that the financial position of the hospital was not a particularly bad one, and in this connection the fact that a legacy of £20,000 was received by the hospital some time ago, while prior to this £10,000 had been raised by a special appeal, no doubt had considerable influence. These receipts certainly reflect credit on the hospital management.

### A WORLD RED CROSS.

The League of Red Cross Societies, as a consequence of the appeal made last year by Mr. A. J. Balfour as Chairman of the League of Nations Council, has undertaken the co-ordination of the work of all national societies or private voluntary organisations at present engaged in arresting disease and relieving distress in the war-stricken areas of Europe. The headquarters of the League are at Geneva, but, in this country, the Imperial War Relief Fund is the League's accredited agent for obtaining the necessary financial support. The national Red Cross Societies of practically all civilised nations have given their cordial indorsement to the scheme, which is mainly intended to prevent duplication of effort on the part of many of the Red Cross Societies concerned and the consequent waste of funds.

### THE LIVERPOOL SOCIAL WORKERS' "HANDBOOK."

The useful work done by the Liverpool Council of Voluntary Aid has been often described in these columns, and the Council's "Quarterly Paper" is a recurrent witness of it. The latest issue mentions an interesting development—namely, a publication to be known as the "Liverpool Social Worker's Handbook." Issued at half-a-crown and published by the Council, the "Handbook" will have two parts. The first deals with the "work of public bodies," and the second with the "work of voluntary bodies." The latter part, beginning with the work of hospitals, dispensaries, and homes, will include sections on infant care, schools for mothers, Personal Service Committee, Police-court work, the unmarried mother, and even a "poor man's lawyer"! At the end will be a Classified List of Voluntary Agencies, which will fill 108 pages. When we contrast the sections of the second part with those mentioned under Part I., "Work of Public Bodies," we pause to wonder which is likely most to concern institutional readers. From the titles of the sections it is impossible to decide. The moral seems to be that the whole volume (it is not sold in parts) should be worth having.

### NEW HOSPITAL AT MOUNTAIN ASH.

Promoted mainly by the miners of the Mountain Ash and Penrhiwceiber district, a movement was set on foot some time ago to acquire Duffryn House, Lord Aberdare's residence at Mountain Ash, for conversion into a general hospital. That project having been abandoned, it is now proposed to secure a plot of land and erect an entirely new hospital at a cost which is estimated at £34,000. There should be little or no difficulty in finding the requisite funds, for the workmen have already accumulated a sum of £16,000, and by the time the building is ready to be opened the balance will doubtless be in the hands of the Committee. Prompted probably by their liability to accident underground, miners show a special eagerness to establish hospitals, and probably half of such institutions in South Wales owe their inception either to the weekly pence or to the inspiration of the miners.

### A HIDDEN HOSPITAL.

The remote institution in the North Riding known as the Hospital of St. John of God, at Scorton, ten miles south-west of Darlington, is little known, so little that the *Yorkshire Post* latterly devoted an article to it. For forty years a fraternity of the Order of the Hospitallers of St. John of God have been treating incurable and chronic diseases ineligible for a general hospital. The patients number at present 100, varying from young men of sixteen to old men of eighty. Some contribute, we learn, 15s. or a pound a week; many nothing. The hospital is supported very largely by voluntary contributions, and has to confess a debt of £375 on the past year. Accommodation appears to be ample. There is room, it is said, for 200 patients, but some wards are empty. One ward received soldiers during the war. The Order, we believe, arose in Portugal about the middle of the sixteenth century. Lyons appears to be its present headquarters, and there are branches in Combres, Croisie (on the Loire), Marseilles, and in Paris, besides others at Leuze (in Belgium), at Dinant, and at Stillorgan (near Dublin). The branch at Scorton, the only English one, was founded in 1881, adjoining the chapel of the nunnery of St. Clare. The present hospital was finished in 1912 at the cost of the Order. The ten Brothers do all the work without the help of women. There is no barrier of creed, no bar to recreation. The difficulty is want of money.

### THE LATEST THING IN SUN-BALCONIES.

The valuable sun-balconies presented by Mr. G. A. Wills, President of the Bristol General Hospital, to the institution have been completed, and embellish the south and west points. They are of steel, the columnar framework and trellis being of "classical design," with Ionic capitals to the pillars. The top tier of these has a glazed roof. The floors of the balconies are also of glass, and thus interfere as little as may be with the light falling on the windows of the wards behind them. Two further balconies have been placed in the inner court of the building for the benefit of the "children's" and the "artisans'" wards. The balconies were designed by the architects, Messrs. Oatley and Lawrence, and have been made by Messrs. Gardiner, Sons and Company, of the Midland Ironworks, Bristol, through the general contractors Messrs. Cowlin and Son, of the same town. Open-air balconies in this country, where the lack is one of sun, not of shade, however useful in themselves, have hitherto tended to sacrifice the free passage of light and air to the wards behind them. The present is an interesting attempt to overcome these difficulties, and we shall hope to hear how far it proves satisfactory, or where, if at all, defects are found.

